# Supporting Innovative Strategies to Reduce Opioid-Related Harms Among Older Adults in Primary Care

**DOI:** 10.1093/geroni/igab046.3222

**Published:** 2021-12-17

**Authors:** Rosanna Bertrand, Nicole Keane, Sarah Shoemaker-Hunt, Clarissa Hsu, Michael Parchman, Sebastian Tong, Arlene Bierman

**Affiliations:** 1 Abt Associates, Abt Associates, Massachusetts, United States; 2 Abt Associates, Cambridge, Massachusetts, United States; 3 Kaiser Permanente of Washington Health Research Institute, Seattle, Washington, United States; 4 Center for Evidence and Practice Improvement (CEPI), Agency for Healthcare Research and Quality (AHRQ), Rockville, Maryland, United States

## Abstract

Older adults are more likely to be prescribed opioids and to suffer from opioid-related harms. Despite growing concerns about opioid misuse in older adults, providers and health care systems often struggle with approaches that would effectively manage opioid use and reduce opioid misuse in older adults. To address this issue, the Agency for Healthcare Research and Quality funded a four-year project to work with primary care practices in developing and testing innovative strategies for opioid management in older adults. To develop a change package that will inform learning collaboratives where primary care practices will be encouraged to test new or modified strategies in managing opioids in older adults, Abt, the contractor, first completed an environmental scan to identify existing resources/tools. Identified resources/tools were vetted by an expert panel and appropriate items were used to develop a change package consisting of nine high-leverage change (HLC) strategies (e.g., Develop processes/workflows that clearly define roles/responsibilities and promote coordinated team-based care). In the change package, multiple key activities that accompany each HLC strategy are presented as examples of strategies that could be implemented to bring about the selected HLC. Primary care practices participating in learning collaboratives will use the change package to guide the development and testing of strategies to manage opioids in their older adults, which will inform the development and refining of a compendium of strategies to best reduce harms of opioid use in older adults.

